# Yeast Mitochondrial Interactosome Model: Metabolon Membrane Proteins Complex Involved in the Channeling of ADP/ATP

**DOI:** 10.3390/ijms13021858

**Published:** 2012-02-10

**Authors:** Benjamin Clémençon

**Affiliations:** INSERM U1055, Laboratory of Fundamental and Applied Bioenergetics, Joseph Fourier University, 2280 Rue de la piscine, BP 53 38041 Grenoble cedex 9, France; E-Mail: benjamin.clemencon@ibmm.unibe.ch; Tel.: +33-476-635-600; Fax: +33-476-514-930

**Keywords:** diffusion, metabolic microcompartmentation, mitochondrial interactosome, phosphotransfer network, metabolon, ATP synthasome, ADP/ATP carrier, VDAC, inorganic phosphate carrier

## Abstract

The existence of a mitochondrial interactosome (MI) has been currently well established in mammalian cells but the exact composition of this super-complex is not precisely known, and its organization seems to be different from that in yeast. One major difference is the absence of mitochondrial creatine kinase (MtCK) in yeast, unlike that described in the organization model of MI, especially in cardiac, skeletal muscle and brain cells. The aim of this review is to provide a detailed description of different partner proteins involved in the synergistic ADP/ATP transport across the mitochondrial membranes in the yeast *Saccharomyces cerevisiae* and to propose a new mitochondrial interactosome model. The ADP/ATP (Aacp) and inorganic phosphate (PiC) carriers as well as the VDAC (or mitochondrial porin) catalyze the import and export of ADP, ATP and Pi across the mitochondrial membranes. Aacp and PiC, which appear to be associated with the ATP synthase, consist of two nanomotors (F_0_, F_1_) under specific conditions and form ATP synthasome. Identification and characterization of such a complex were described for the first time by Pedersen and co-workers in 2003.

## 1. Introduction

An essential step in the determination of structure-function relationships at a molecular level is to investigate the intrinsic properties of isolated proteins, via molecular biology, biochemical and structural approaches [1–3]. However, it is also necessary to study protein function in an integrated system in order to understand the phenomena in cellular and physiological contexts. Energy metabolism is an example in bioenergetics, which operates as an integrated network of molecular interactions, where the spatial and temporal organization of each of these cellular compounds is crucial. It is especially important, if one takes into account the high degree of complexity of eukaryotic cells and the diversity and abundance of proteins. In addition, all membranes, which macrocompartmentalize the cytosol [4] and cytoskeletal network [5], are barriers to the metabolite diffusion as well as the density of the cytoplasmic macromolecular complexes [6]. Indeed, the concentration of macromolecules in the bacterial cytoplasm is about 300–400 mg/mL [7] representing 30% of the total volume. In the case of the mitochondrial matrix of eukaryotic cells, it amounts to 60% [8]. This organization and the intracellular macromolecular crowding result in restriction of metabolite diffusion [9], creating confined spaces named microcompartments isolated from the rest of cellular processes [10]. This results in an increase of the local protein concentrations altering the equilibrium constants by promoting the association of the macromolecules with each other [11] to form multi-protein [12] or protein-DNA complexes [13]. Enzymatic reactions with small molecules are favored [14]. Overall this plays a key role in protein folding [15,16] and conformational changes [17,18]. It is easy to understand the fundamental role of the intracellular complex environment on the major cellular functions and the stakes of a holistic approach with molecular biology systems. The microcompartments, involving the concepts of multi-enzyme complexes and metabolic channeling, are the basis of the organization and regulation of cellular energy metabolism [19–21]. Indeed, the transfer of substrates is carried out through interconnected metabolic pathways, either between two enzymes via random diffusion according to the Einstein-Smoluchowski equation [22] or by a direct transfer [23,24]. These functional couplings allow the accumulation of products and reaction intermediates in a confined space. The importance of such phenomena lies partly in the fact that physically associated enzymes or transient multi-enzyme complexes have the potential to exhibit unique catalytic properties in contrast to isolated enzymes [25,26]. Compartmentalization may include both metabolites and enzymes. The enzymes catalyzing sequential reactions in given metabolic pathways have been proposed to be highly organized in supra-molecular complexes named “metabolon” by Paul Srere [27–29]. They are involved in signaling cascades or metabolic pathways such as glycolysis [30–34], of which the enzymes can be linked to the cytoskeleton [35–39], tricarboxylic acid cycle [40], lipid and amino acid metabolisms [41,42], or protein biosynthesis [43]. The most representative example of functional coupling was described for the first time by Pedersen *et al.*, in the case of myocardial cells in which the mitochondrial creatine kinase (MtCK) located in the intermembrane space is coupled to the ATP synthasome [44]. The ATP synthasome corresponds to a super-complex located in the inner mitochondrial membrane and comprising the ADP/ATP carrier, the inorganic phosphate (Pi) carrier and the F_1_F_0_-ATP synthase. It is driven by the proton electrochemical gradient [45,46], which is generated by the respiratory chain to catalyze ATP synthesis from ADP and Pi. This biological phenomenon achieves a functional coupling, which avoids an entropy increase. It is reminiscent of the thermodynamic properties associated with a philosophical concept, which was subject to much debate in the history of physical science, because it contradicts the second law of thermodynamics, namely Maxwell’s demon theory [47]. One of the clearest pieces of evidence for such a functional coupling comes from the measurement of the apparent *K*_M_ of the ADP/ATP carrier for ADP, which provides information about the availability of ADP for oxidative phosphorylation (OxPhos). Indeed, it was noted that the value *K*_M_ measured with isolated mitochondria is about 10 μM, depending on organisms. However, *in situ*, this value is 20-times higher in myocardial fibres [48], reflecting mainly a slower ADP diffusion. The addition of creatine (Cr) reduces the *K*_M_ value, highlighting the role of the functional coupling of creatine kinase (CK) and the ADP/ATP carrier in energy metabolism.

The aim of this review is to provide a detailed description of the different partners involved in synergistic ADP/ATP transport across the mitochondrial membranes in the yeast *Saccharomyces cerevisiae* and to describe on the basis of detailed literature analyses what could be a mitochondrial interactosome.

## 2. The Different Proteins Involved in the Mitochondrial Membrane Transport of Adenine Nucleotides

### 2.1. The ADP/ATP Carrier

#### 2.1.1. Overview

**Figure 1:**
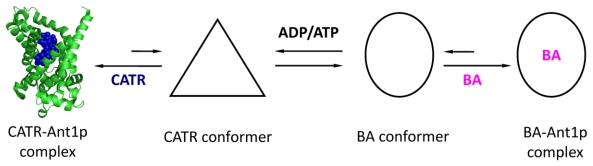
Conformational states adopted by the ADP/ATP carrier during the nucleotides transport. carboxyatractyloside (CATR) and bongkrekic acid (BA) inhibit the transition by locking the carrier in stable complexes.

A member of the Mitochondrial Carrier Family (MCF), the mitochondrial ADP/ATP carrier (Aacp) fulfills the cellular energetic needs by exchanging the neo-synthesized matrix ATP for the cytosolic ADP. In 1965, E. Pffaf discovered a specific exchange of adenine nucleotides through the membranes of mitochondria isolated from rat liver and demonstrated the existence of a specific carrier [49]. Later, other groups showed that this carrier was a protein [50]. The name of mitochondrial ADP/ATP carrier was then proposed [51]. This protein plays a very important physiological role in the renewal of cellular energy. Indeed, a human adult renews his own weight of ATP per day and a large proportion of it passes through the ADP/ATP carrier. In spite of this, Aacp has a low transport activity, *i.e.*, about 1000 to 2000 min^−1^ at 20 °C in mitochondria isolated from rat heart [52,53]. This low rate of transport is physiologically balanced by the abundance of the protein in the mitochondrial membranes and its functional coupling. In certain tissues, Aacp may represent almost 10% of the proteins of the inner mitochondrial membrane. Under physiological conditions, one cytosolic ADP^3−^ molecule is exchanged against one matrix ATP^4−^ molecule, both in the form of free nucleotides each carrying 3 or 4 negative charges, respectively. Therefore, this exchange is electrogenic and its direction is driven by the membrane electrochemical potential created by the respiratory chain [54,55]. In the absence of this potential, the ADP/ATP carrier exchanges either ADP^3−^ or ATP^4−^ against one or another according to the concentration gradient [56]. The discovery and characterization of Aacp are related to the finding of specific inhibitors belonging to two families: atractyloside (ATR and its *in vivo* biological precursor, the carboxyatractyloside (CATR)) and bongkrekic acid (BA). It has been established that CATR and BA can recognize distinct pre-existing carrier conformations, commonly known in the literature as “CATR conformer” and “BA conformer”. Both conformations are stabilized by inhibitor binding and represent extreme states adopted by the carrier during the ADP and ATP translocation process. This transport mechanism has been extensively studied thanks to CATR and BA (Figure 1). The structure of isoform 1, of the bovine ADP/ATP carrier (Ant1p) in complex with CATR, was solved in 2003 at high resolution [57]. It is characterized by a wide cavity open to the intermembrane space, which is probably involved in the transport mechanism. CATR is located deep inside and interacts with residues R79, N87, K91, R187, R234 and D231. On the assumption that CATR and ADP binding sites overlap, at least partially, some interesting predictions were made [58]. The Uncoupling Protein (UCP2) structure, a MCF member, was recently solved and both proteins exhibit similar organization: six transmembrane segments delineating the cavity, open towards the cytosol and three large matrix loops [59]. However, UCP2 is less tightly closed on the matrix side than the ADP/ATP carrier. Despite this outstanding progress, biochemical data currently available do not give insights into the precise molecular mechanism of ADP and ATP translocation across the inner mitochondrial membrane.

#### 2.1.2. Oligomeric State of the ADP/ATP Carrier

In 1975, the first data on the possible multimerization of the ADP/ATP carrier was published [60]. Analyses of the CATR/protein stoichiometry were in favor of one mole of inhibitor bound to two moles of the ADP/ATP carrier, suggesting a dimeric organization of the carrier. These results were later confirmed by other physico-chemical and biophysical analyses.

Analytical centrifugation and small-angle scattering experiments suggested a dimeric organization of the protein in detergent micelles [61–63]. In addition, experimental evidence for a dimeric organization of other MCF members, such as the oxaloglutarate, citrate, or Pi carriers, support the hypothesis of an oligomerization of the mitochondrial carriers [64,65]. A tetrameric functional unit of Aacp was also suggested because two nucleotide binding sites on each side of the carrier and of different affinities were characterized for one transport unit [66,67]. However, recent findings questioned the existence of an oligomeric state of MCF members. The high-resolution structure obtained for the CATR-Ant1p complex shows a single monomer bound to a molecule of CATR. Later on the authors obtained another structure where a single monomer is in contact with another monomer through cardiolipins [68]. However, the crystallographic dimer appears artefactual and is contradictory to previously published data because it contains one molecule of CATR per monomer. Neverless, recent experiments based on analytical centrifugation with improved in system analyses and data processing, confirm the results published in the early 80’s. These data also suggest that if Ant1p forms predominantly monomeric complexes with CATR in Triton X-100 solution, it can also form multimeric complexes [69]. A monomeric organization of the yeast Aacp was recently claimed through the use of various biochemical or biophysical approaches [70–72].

#### 2.1.3. The ADP/ATP Carrier of *S. Cerevisiae*

**Figure 2:**
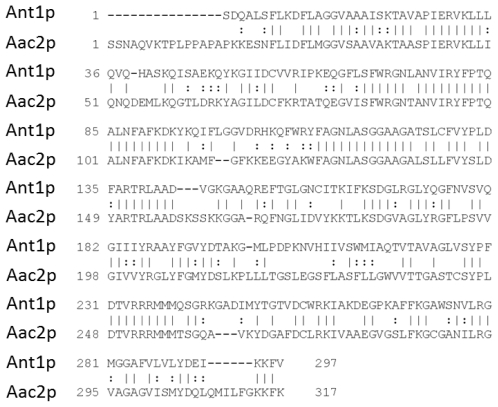
Primary sequence alignment of bovine ADP/ATP carrier (Ant1p) and ADP/ATP carrier (Aac2p). Sequence alignment was performed with the *Needle* program. The bars show the identical amino acids and the two points the similar residue. Numbering refers to the Aac2p sequence. Both sequences are 49.5% identical.

Studies of the ADP/ATP transport were conducted for many years with *S. cerevisiae* thanks to the ease of genetic engineering of this organism [73]. *S. cerevisiae* has three isoforms named Aac1p, Aac2p, and Aac3p, with different physiological characteristics. Isoform 1 is encoded by the *AAC1* gene on chromosome XIII in yeast. Aac1p is expressed weakly and constitutively under aerobic conditions and repressed in the presence of oxygen [74]. The second isoform is encoded by the *AAC2* gene on chromosome II and is highly expressed under aerobic conditions in the presence of a non-fermentable carbon source such as lactate [75]. Aac2p is necessary for yeast growth on a non-fermentable carbon source and therefore represents the homologue of the human Ant1p, sharing approximately 50% of sequence identity [76] (Figure 2). Because of its high expression and its involvement in energy production during respiration, the second ADP/ATP carrier isoform of *S. cerevisiae* has been the most studied. The third isoform is encoded by the *AAC3* gene whose expression is repressed in the presence of oxygen [77,78]. The three genes encode transporters of 309, 318 and 307 amino acids, respectively. When overexpressed, *AAC1* and *AAC3* can complement an *aac* null strain.

#### 2.1.4. Human Pathophysiological Aspects

Four human genes named *ANC1/ANT1*, *ANC2/ANT3*, *ANC3/ANT2* and *ANC4*/*ANT4* encode for the ADP/ATP carrier in different tissues. Isoform 1 is expressed in the heart and skeletal muscles, brain and organs with low mitotic regeneration. Isoform 2 is found weakly throughout the human body. The third isoform is highly expressed in growing cells, with high energy needs, such as the kidneys, liver, spleen but also in cancer cells [79,80]. Finally, the fourth isoform has been identified from a collection of expressed sequence tags and this transcript was localized in the testis, liver and brain [81]. The direct involvement of human ADP/ATP carrier mutants was described in five cases of ophthalmoplegia and one case of cardiomyopathy [82].

### 2.2. The Inorganic Phosphate Carrier—PiC

The mitochondrial phosphate carrier (or Phosphate Transport Protein or p32) catalyzes the transport of Pi into the mitochondrial matrix where it is mainly used for OxPhos [83–86] and especially for the ATP biosynthesis. Like the ADP/ATP carrier, this transporter is essential for cell development of strictly aerobic organisms.

#### 2.2.1. Discovery and Biochemical Properties of PiC

The bovine PiC carrier and the associated gene (*PIC*) were discovered in the mid-80’s [87,88]. The *PIC* gene was subsequently sequenced, cloned and studied in the yeast *S. cerevisiae* [89]. A bi-functionality was described and was then the matter of a long controversial debate. Its secondary hypothetical role as an import receptor for nuclear-encoded preproteins into mitochondria was disproved by Zara’s group who stated the alone function of PiC in the translocation of Pi [90]. This carrier is a MCF member, with six transmembrane segments [91,92], but remains poorly characterized compared to Aacp.

#### 2.2.2. Transport Mechanism of Mitochondrial PiC

The Pi carrier catalyzes the electroneutral transport of phosphate through the inner membrane from the intermembrane space to the mitochondrial matrix but the involved mechanisms are poorly understood. Nevertheless, a passive mode of transport using the proton gradient driven by membrane potential (Δ*Ψ*) was proposed. The PiC would work as a Pi/H^+^ symporter [93,94] or alternatively as a Pi/OH^−^ antiporter [95]. It was also suggested that the Pi can be transported only as monocharged ion [96]. With regards to the mechanism of transport, some experiments show that PiC of *S. cerevisiae* can switch to a uniport mode after being modified at cysteine 28 in the presence of mercury (HgCl_2_) [97]. Finally, PiC is inhibited by fatty acids, particularly the 12-(4-azido-2-nitrophenylamino) dodecanoic acid (AzDA) [98] and can be photolabeled by fatty acid derivatives bearing an azido-nitrophenyl group. Thus, the authors proposed that this carrier may also be involved in the metabolism of fatty acids.

Biochemical studies of the yeast Pi carrier were carried out after exogenous expression in the form of inclusion bodies in *Escherichia coli* [99]. Functional studies of the yeast PiC showed that N-ethylmaleimide (NEM) could specifically target cysteine 28 of the carrier with the stoichiometry of one NEM per PiC subunit to inhibit the transport of Pi. Studies of cysteine mutants of the yeast Pi carrier expressed from inclusion bodies were subsequently performed [97]. The cysteines at position 28, 134 and 300 were replaced one by one with serines. The results initially showed that cysteine 300, near the C-terminus and cysteine 134, located in the third transmembrane segment, are accessible to the hydrophilic reagent butyl-SH, in contrast to cysteine 28 localized in the first transmembrane segment. This binding to the reactive cysteine 134 results in complete inhibition of the transport. The reversible conversion capacity of PiC to form a uniport mode of transport to an antiport appears to be dependent only on cysteine 28. The PiC of *S. cerevisiae* expressed in inclusion bodies in *E. coli* may be partially resolubilized in the presence of sarkosyl detergent and is monomeric or unstructured. After exchanging sarkosyl with a polyoxyethylene and dialysis, PiC is organized in stable and functional dimers [100]. Although it is accepted that Aacp has common features with PiC, there is currently no biochemical evidence for a monomeric organization of PiC unlike with Aacp [101]. Mayr and co-workers [102] described patients with mitochondrial phosphate carrier deficiency. A young adult presented cyanosis and muscular hypotonia. A hypertrophic cardiomyopathy was detected with low cardiac output and elevated levels of lactate in plasma. Severe muscular hypotonia was also observed.

### 2.3. The Mitochondrial Porin

In contrast to the high selectivity of the inner membrane, due to the presence of specific carriers as Aacp or PiC, the outer membrane is more permeable and plays a key role as a molecular filter, retaining molecules greater than 3000–5000 Da [103]. This controlled diffusion of metabolites occurs through transmembrane channels present in large amounts in all eukaryotes: the mitochondrial porins. Thus, in the case of the yeast *S. cerevisiae*, analyses carried out after purification of the porin isoform 1 (denoted Por1p, the most abundant isoform) show that this protein represents at least 10% of the total proteins of the mitochondrial outer membrane [104] and up to 50% in *Neuraspora crassa* [105].

#### 2.3.1. Identification and Physiological Characteristics of Porin

The first studies on the biochemical composition of outer membrane proteins in different eukaryotes confirmed a major protein with a molecular mass of about 30 kDa with an unknown function [105]. In 1976, it was identified in the unicellular organism *Paramecium aurelia*. After that, it was reconstituted in an artificial membrane, and its electrophysiological characteristics were described [106]. It was then named VDAC for “Voltage-Dependent Anion Channel”. Subsequently, this porin was identified in rat, beef, *S. cerevisiae*, *N. crassa* [107] and in other eukaryotic organisms, with highly conserved biochemical and biophysical properties [108].

The electrophysiological experiments conducted on the reconstituted porin in liposomes or planar lipid bilayers show that the VDAC controls the metabolites flow through changes in the frequency of channel opening following membrane potential [109]. Thus, there is a condition called “open” state, where porin is slightly selective to anions. It is characterized by high permeability and the presence of large water-filled pores (2–3-nm diameter), observed by transmission electron microscopy [110].

Conversely, when the membrane potential difference is greater than 30 mV, it is possible to distinguish another state called “closed”, in which the permeability is reduced and does not allow the transport of anions such as ADP^3−^ or ATP^4−^. It was shown *in vitro* that the opening of the channel is regulated by various molecules, such as glutamate and NADH [111]. Although these electrophysiological experiments afford a convenient test for the mitochondrial porin functionality revealing molecules that influence the opening or closing of the channel, it is important to remember that, *in vivo*, the outer mitochondrial membrane is not subjected to a membrane potential due to its permeable nature, although some works attest to the contrary [112–114]. Depending on the organism, there may be multiple isoforms of VDAC and it is interesting to note that the more complex the organism, the higher the number of isoforms. Thus, there is only one isoform in *N. crassa* while in mice and humans three isoforms exist. In the yeast *S. cerevisiae*, there are two isoforms of porin. The most abundant, and therefore the most studied one, is the isoform 1 (denoted Por1p) which is encoded by the nuclear gene *POR1* [104]. Isoform 2 (denoted Por2p) is encoded by the gene *POR2* and is 50% identical to Por1p [115]. In contrast to the latter isoform, Por2p is weakly expressed in mitochondria, regardless of the culture conditions, and exhibits a low permeability to metabolites [116]. Contrary, to Aacp and PiC, VDAC is not essential for the yeast growth on a non-fermentable carbon source. It was assumed that the facilitated diffusion of small molecules across the mitochondrial outer membrane is mediated exclusively by the porin. However, the viability of the *Δpor* mutant suggests the existence of another pathway for metabolite exchange between mitochondria and cytosol [117,118].

#### 2.3.2. High-Resolution 3D Structures of Porin

**Figure 3:**
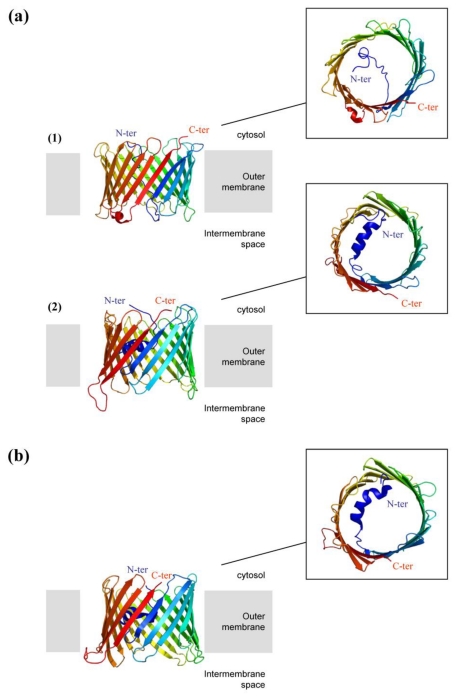
Structure of mitochondrial porins (**a**) Isoform 1 of human porin obtained by NMR spectroscopy [120] (1) and by an approach combining both NMR spectroscopy and X-ray crystallography [119] (2); (**b**) High-resolution structure of an isoform of the murine Voltage-Dependent Anion Channel (VDAC1) solved by X-ray crystallography [121]. All these structures exhibit a channel formed by 19 stranded antiparallel β-sheets in the transmembrane core protein. The N-and C-terminal ends are oriented towards the cytosol. These structures classify protein VDAC in a new family of porins. The N-terminus is an organized structure (α-helix) or not, whose orientation differs from one model to another.

By examination of its 3D structure, porin can be divided into two parts. The core of the protein corresponds to the relatively mobile transmembrane channel composed of 19 antiparallel β-sheets. The “soluble” N-terminal extension is twenty amino acids long, folded into an α-helix and connected to the β barrel by a hinge peptide. The high-resolution 3D structures of human and murine VDAC1 porins (hVDAC1 and mVDAC1) were recently determined (Figure 3) [119–121]. Each of them was obtained using a similar approach based on the exogenous expression of VDAC1 within inclusion bodies in the bacteria *E. coli*. This requires refolding of VDAC in the presence of detergent. The aim was to obtain large amounts of protein, which is a preliminary requirement for X-ray crystallography and NMR spectroscopy (Nuclear Magnetic Resonance). Hiller and colleagues were the first to elucidate the structure of the porin hVDAC1 at high resolution, in the detergent LDAO (Lauryldimethylamine-oxide), by NMR spectroscopy [120]. Bayrhuber and colleagues used an approach combining NMR spectroscopy and X-ray crystallography to obtain the porin structure in Cymal-5. However, it was resolved at a lower resolution (4 Å) [119]. Finally, Abramson’s team determined the crystal structure of the murine VDAC1 solubilized in the presence of the lipid (1,2-dimyristoyl-sn-glycero-3-phosphocholine) and the detergent CHAPSO and resolved it at high resolution (2.3 Å) [121]. It is interesting to note that the three final structures are relatively similar. Indeed, the human and murine porins are organized in a barrel consisting of 19 stranded antiparallel β-sheets with the N-and C-termini pointing towards the cytosol, classifying the mitochondrial porin in a new family of porins [120]. One of the major differences between these structures is the N-terminal α-helix, which was described in two of the three structures solved. Its orientation within the channel is a matter of debate (Figure 3). The reason for this difference is unclear but may potentially be related to the experimental conditions: histidine tag position, type of detergent used, and pH of refolding. Nevertheless, these results are consistent with the idea that the N-terminal end may be involved in the mechanism of opening and closing of the pore via its motion. Indeed, in the structure described by Hiller, the N-terminal segment divides the lumen into two parts [120]. It was then suggested that it corresponds to a closed state since the pore diameter is reduced compared to the other structures of VDAC. In contrast, in the crystal structures of mVDAC1 and hVDAC1 porins, the N-terminus is located horizontally against the inner wall of the barrel and is parallel to the membrane plane [119,121]. This location has two consequences: (i) the pore diameter appears larger, (ii) the electrostatic distribution is changed in favor of a global positive charge. It was therefore suggested that these structures probably correspond to states of the anion-selective pore open towards the cytosolic side. The importance of the N-terminal domain in the mechanism of pore opening and closing is still under debate. However, complementation studies were undertaken with human cells of which the endogenous VDAC was knocked down by siRNA. A mutant mVDAC porin, lacking its first 26 amino acids, was expressed in these cells which presented the same characteristics as wild type cells [122]. This surprising result suggests that the N-terminus is not essential for the main VDAC function, which is transporting small solutes. However, the authors showed that the N-terminal domain of VDAC is an essential mediator in apoptosis. Indeed, this domain is involved in the release of cytochrome c in the cytosol and in the recognition of anti-apoptotic proteins such as hexokinase and Bcl2.

Although, some aspects are still controversial, the 3D structures of VDAC recently obtained are a major asset in understanding the molecular mechanisms involved in the transport of small metabolites such as ADP, ATP and Pi through the outer membrane of mitochondria. This mechanism seems now to be generating major interest. However, since their discovery, these NMR and X-Ray determined 3D structures are still the subject of relentless and heated debate, which is not mentioned in this review. In fact, disagreement arises when it comes to determine the exact topology of VDAC in the membranes as well as the number and orientation of the β-sheets that form the barrel. According to Colombini, these 3D structures are not consistent with previous functional data. This author suggests that none of them correspond to the native structure. Instead, he proposes a functionally-derived structure of the pore composed of one α-helix and 13 β strands tilted at a 46° angle with an internal diameter of 2.5-nm [123]. The gating mechanism of the voltage-dependent anion channel implies the existence of one or more voltage-sensor domains. Indeed, Colombini proposes a model of a voltage gating process involving a mobile positively charged voltage-sensor domain, whose position is driven by the membrane potential. This large conformational change was deduced from porin properties of conductance, pore size, selectivity and pore volume [123]. Thus getting a good fit between functional and structural data remains very challenging for the porin but also for numerous other proteins.

#### 2.3.3. Selectivity of the Pore

With regard to the transport of metabolites, VDAC has long been considered as an ordinary molecular filter whose selectivity cannot be reduced to its pore diameter. On this principle, all molecules with a steric bulk adapted to the size of the pore could potentially cross through. However, today this definition seems outdated. Indeed many studies suggest that the mitochondrial porin has some selectivity since several binding sites and protein partners have been identified. Thus the protein seems to have a much larger dimension than previously thought, due to its involvement in major cellular functions (channels energy, cell death, *etc*.).

##### 2.3.3.1. Nucleotide Binding and Derived Molecules

It was previously suggested that the VDAC porin is regulated by adenine nucleotides and their derived molecules [124–126]. In the case of *N. crassa* porin, it was shown that NAD(P)H, ATP and ADP regulate the diameter of the pore and the existence of ATP binding sites (NBS noted for “Nucleotide-Binding Sites”) has been proposed [127–130].

The presence of NBS in mammalian VDAC porins was confirmed by their binding to ATP agarose columns [124]. In addition, interaction between ATP and porin was demonstrated by binding experiments with radiolabeled ATP (noted [^32^P]-ATP) [124]. More recently, Yehezkel and colleagues demonstrated ATP binding at low and high affinity sites of mVDAC1 using a photoactivable analogue of ATP (benzoyl-benzoyl-ATP noted [α-^32^P]-BzATP) [131].

MALDI-ToF-MS analyses of porin, labeled with [α-32P]-BzATP, confirmed the binding of two molecules of BzATP per porin molecule. The sites are located in the C-terminal (271–283) and N-terminal (19–25) regions of the porin [131]. Studies by site-directed mutagenesis supplemented these results by showing the importance of lysine 20 in the binding of mVDAC1 to ATP [132].

##### 2.3.3.2. Binding of Ca^2+^

Ca^2+^ plays a major role in the cell by creating a link between the mitochondria and the cytosol. In point of fact, calcium is primarily an intracellular messenger in signal transduction [133–135]. The intramitochondrial Ca^2+^ concentration modulates the activity of critical enzymes of the Krebs cycle, oxidation of fatty acids, catabolism of amino acids, ATP synthase and Aacp.

In the mammalian mitochondria, elevated matrix Ca^2+^ concentrations (over 1 to 3 μM) induce an increase in the permeability of the inner membrane caused by the opening of a nonspecific pore (“Permeability Transition Pore” or PTP) [136,137]. It was shown that the hVDAC1 is permeable to Ca^2+^ and exhibits binding sites, which control its activity [138]. They were identified through the use of a photoactivatable reagent, azido ruthenium (AzRu), which is able to interact specifically with Ca^2+^ binding proteins and accordingly significantly inhibits activity. MALDI-ToF-MS analyses of AzRu-labeled mVDAC1 reveal the presence of two Ca^2+^ binding sites corresponding to residues E72 and E202 [139].

##### 2.3.3.3. Potential Partners of the Porin

Table 1 summarizes the main protein partners interacting with the VDAC and involved in different cellular functions. They are mainly hexokinase [140–143], Aacp [144–146], MtCK [147–150], pro-apoptotic proteins belonging to the Bcl2 family [151–154], tubulin (described as factor X) [155–158] and IP_3_ receptor [159].

In *S. cerevisiae*, the interaction of tubulin with VDAC is not obvious since it is known that the mitochondrial dynamics are driven primarily by interactions with the actin cytoskeleton [160]. The interaction domains characterized or hypothesized are presented in Table 1.

#### 2.3.4. Supra-Molecular Organization of Porin in the Membrane

The oligomeric organization of porin is also discussed. Though some studies concluded that VDAC exists as a functional monomer, which was recently crystallized [121], other data in particular blue native PAGE analysis claim that structures of rat and human porins in the membrane correspond to dimeric, trimeric, tetrameric and hexameric architecture [161–167]. However, the reasons for an oligomerization of porin are not completely understood, given that the channel through which metabolites pass occupies the center of the barrel and the formation of an oligomer does not appear as necessary for the pore to operate. It was therefore proposed that the supra-molecular organization of porin has a role in stabilizing the protein. This could be also true for the bacterial porins [168]. A recent paper describes the mVDAC1 porin in a dimeric architecture where both monomers are oriented in the opposite direction [121]. The hexamer arrangement of porin dimers oriented head to tail in the crystals could possibly mimic the native organization observed in electron diffraction studies [161] and more recently in AFM (Atomic Force Microscopy) experiments with the mitochondrial outer membranes [167].

#### 2.3.5. Role of Lipids in Porin Activities

Sterols are present in large quantities in the mitochondrial outer membrane, with five cholesterol molecules per monomer of porin in bovine mitochondria [169]. In the case of *N. crassa* [170,171] and *S. cerevisiae* [172], large amounts of ergosterol were detected in preparations of porin. The importance of sterols for the VDAC function was shown *in vitro* [173]. Indeed, *Dictyostelium discoideum*, *Paramecium* and rat porins, once delipidated, are unable to form functional channels in artificial membranes. However, simple pre-incubation of these proteins in the presence of sterols and detergent allows them to regain the ability to transport. This suggests that sterols are essential to the proper functioning of porin in the mitochondrial outer membrane. However, these results can be viewed with caution since, in the case of the mammalian ADP/ATP carrier reconstituted into liposomes, the nucleotide exchange activity is stimulated in the presence of cholesterol while the same sterols are absent from the inner mitochondrial membrane [174]. The effect of sterols on the function of these two proteins could be indirect, and caused by the change in composition, and thus of the physical state, of the artificial membrane used for reconstitution, and not by a specific interaction between the *in vivo* sterols and the membrane protein of interest. Other lipids seem to play a role in voltage gating of the pore, such as non-lamellar lipids of the mitochondrial outer membrane [175].

## 3. Mitochondrial Interactosome Model

**Figure 4:**
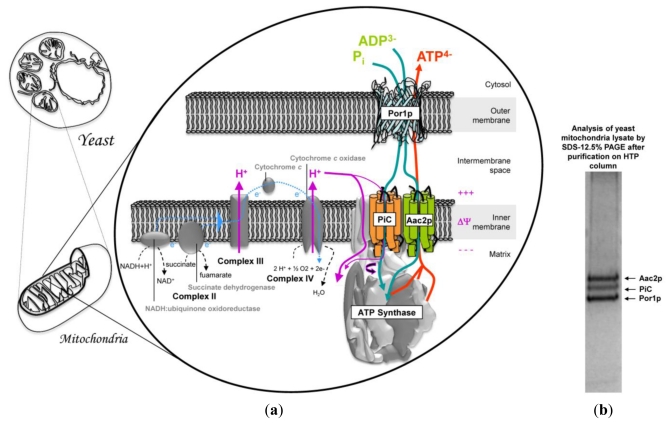
Machinery model of active transport driven by the electrochemical gradient of protons across the mitochondrial membranes. (**a**) The inorganic phosphate (Pi) and ADP^3−^ are imported into the matrix via two carriers: Aac2p and PiC while the matrix ATP^4−^ is consumed by the ATP synthase, shown in its monomeric form. The charge of each transported molecule is indicated. The membrane potential is negative in the matrix. The outer membrane ensures free passage of these compounds though the VDAC, noted Por1p; (**b**) Gel electrophoresis showing the co-purification of Aac2p, PiC and Por1p from a yeast mitochondria lysate in n-dodecyl-β-D-maltoside/emulphogen mix (DDM/EM) after chromatography on a hydroxylapatite column (Coomassie Blue staining of the SDS-PAGE gel).

Mitochondrion plays a key role in cellular functions. Indeed, it is involved in apoptosis, thermogenesis, calcium homeostasis, and in many anabolic pathways such as heme synthesis, protein in iron-sulfur clusters, nucleotides or steroids. Above all it plays a fundamental role in oxidative catabolism leading to the production of a usable form of energy, ATP. ATP synthesis is not the only process driven by the electrochemical proton gradient. Indeed, in mitochondria, many small charged molecules such as ADP and Pi are transported into the matrix from the cytosol, while others, such as ATP, must be transported in the opposite direction (Figure 4). Membrane proteins that realize the transport of these molecules can couple their transition from the inner membrane to the mitochondrial matrix with the energetically favorable flow of protons. For example, Pi is co-transported with a proton by PiC from the intermembrane space into the matrix. In contrast, ADP is transported against ATP by Aacp. As the ATP molecule has an additional negative charge compared with ADP, each nucleotide exchange is accompanied by the movement of a negative charge from the inside to the outside of the mitochondrial matrix. This ADP/ATP antiport is facilitated by the potential difference between both sides of the inner membrane. Finally, it has been suggested that passaging of adenine nucleotides and also Pi across the mitochondrial outer membrane is mainly carried out by the VDAC.

The biochemical properties of the main actors of this transport machinery, Aacp, PiC and Por1p have been previously described. It is important to note that the model proposed in Figure 4 applies only to yeast, which lacks MtCK (www.yeastgenome.org/).

Indeed, in higher organisms, particularly in myocardial cells, the MtCK catalyzes the transphosphorylation of creatine (Cr) to phosphocreatine (PCr) from the hydrolysis of ATP, newly synthesized in the mitochondrial matrix [176,177]. This enzyme forms an octamer, which is localized in the intermembrane space and at the level of mitochondrial cristae [178,179]. As for Aacp, it was shown that MtCK interacts with membrane receptors, which are negatively charged phospholipid cardiolipin and specifically found in the inner membrane [179–181]. Other studies suggest that MtCK interacts with phospholipids of the outer membrane and with the VDAC porin [150]. A model in which MtCK is associated with VDAC and Aacp was proposed to ensure the “channeling” of ATP through the mitochondrial membranes and, at the same time, this would contribute to the formation of mitochondrial contact sites [182]. The functional coupling of MtCK with ATP production has been demonstrated for the first time in the muscle by biochemical, kinetic, thermodynamic and radioisotopic analyses.

Finally, some contact sites, comprising, among others, the VDAC and the ADP/ATP carrier [183] would help in stabilizing the mitochondrial creatine kinase [184]. They are expected to play an important role in energy metabolism and cell apoptosis [185]. This model is quite speculative as it is proposed by combining data from literature taken independently. However there is currently no evidence of direct interactions between these proteins, with the notable exception of the work done by Claypool and co-workers [186]. This group was interested in defining the yeast ADP/ATP carrier interactome. Their results demonstrate a potential interaction, mediated by the presence of cardiolipin (CL) associated to Aac2p, between Por1p and PiC but also with subunits of the respiratory complexes III and IV. Nevertheless, it is interesting to note that these three proteins were co-purified by chromatography on a hydroxylapatite column (HTP Bio-Rad) from a lysate of yeast mitochondria in DDM/EM (dodecyl-β-d-maltoside/emulphogen mix) [187]. Unfortunately, there is no clear evidence of stable protein complexes, because mitochondrial dynamics requires some plasticity for mitochondrial organization. One hypothesis is probably that of an effect of external constraint that would promote the existence of this super-complex through lipids. Indeed, the interactions between membrane proteins and lipids are essential to many fundamental cellular processes such as respiration, photosynthesis, transport of molecules, signal transduction and cell motility. Progress in addressing structural membrane proteins has revealed the presence of strongly associated lipids which are often located in very specific sites [57,188]. The binding of lipids gives to these proteins some structural stability, which can affect their folding or insertion in the cell membrane [189,190]. This model is probably more complex, if we take into account the data from the literature that demonstrate co-purification of Aac2p with another component of the OxPhos, the cytochrome *bc*_1_-COX super-complex (composed of cytochrome *c* reductase and cytochrome *c* oxidase) and its association with the TIM23 machinery [191]. In addition, the OxPhos and the cytochrome *bc*_1_-COX are located in the inner membrane and the dimeric ATP synthase in cristae. A new super-complex comprising these proteins was recently characterized [192–194]. It was proposed that it intervenes directly in the architecture of mitochondria, creating contact sites at the level of cristae junctions. The formation of these contact sites is mediated by mitofilin (Fcj1), a hydrophobic protein located at the intermembrane space and anchored by its N-terminal domain in the inner membrane. Fcj1 belongs to MINOS (Mitochondrial INner membrane Organization System) or MitOS (Mitochondrial-Organizing Structure) supercomplexes containing some other components: Aim 5, Aim 13, Aim 37 and Mio 10 (Mos1), Mio 27 (Mos2). As a conclusion, mitofilin plays a key role in organizing the microcompartimention in the intermembrane space of mitochondria and therefore in the metabolism. In addition, coupled with the outer membrane via the TOM complex (Tom40-Tom22), it is involved in the import of proteins.

## 4. Conclusions

In yeast, the putative mitochondrial super-complex is different from the one proposed in mammalian cells for various reasons. The main one is the absence of mitochondrial creatine kinase. Especially in the myocardial cell, MtCK plays a role in determining the functional transport of mitochondrial ATP to the rest of the cell. Its absence in yeast could be offset by a strong spatial promiscuity of the various partners of this protein machinery (PiC, Aac2p and Por1p), described in the literature as mitochondrial contact sites. In addition, the absence of biochemical and biophysical evidence for the existence of physical interactions of these proteins suggests the super-complexes are probably glued by lipids specifically found in the mitochondrial membranes, such as ergostreol and cardiolipin. This interactosome would be a super-complex forming a microcompartment with particular biochemical properties and efficiently providing the ATP for the major cellular functions. This molecular organization will probably appear more complex since the probable oligomerization of mitochondrial ATP synthase dimers in yeast will be deciphered [195]. Finally, the existence of such a transient complex would be consistent with the dynamic nature of the mitochondrial compartment. Indeed, in addition to its shape variation, the mitochondrial network is constantly being reshaped by the phenomena of apparent balance of fusion and fission that determines the arrangement of mitochondria in the cell [196].

## Figures and Tables

**Table 1:** The potential partners of the mitochondrial porin.

Protein partners	Interaction partners domain	Mediators		Interaction VDAC * domain		Roles	Protein partners	References
		activators (+)	inhibitors (−)					
**Hexokinase I and II (HK I and HK II)**	N-terminal hydrophobic α-helical (MIASHLLAYFFTELM)	Mg^2+^, Residues E188 and E202 of VDAC	Glucose-6-phosphate (G6P) and DCCD *	**Amino acids**	**Location in the structure**	PTP *	Mitochondrial apoptosis	
				E72	β sheet n°4			[140–142]
				E65	Cytosolic loop n°2			[143]
				D77	Matrix loop n°2			
				K73	β sheet n°4			
Aacp*	Cardiolipin-induced							[144–146]
MtCK*	Cardiolipin-induced							[147–150]
**Bcl2 family proteins**	H5 and H6 transmembrane helix	-		-		PTP	Mitochondrial apoptosis	[151–154]
**Tubulin**	Tubulin anionic C-terminal tail (CTT) peptides	-		VDAC lumen		regulation	Mitochondrial respiration	[155–158]
**Inositol 1,4,5- triphosphate receptor (IP_3_R)**	N-terminal domain	Chaperone grp75		-		Scaffolding the ER *-mitochondria contacts	Ca^2+^ uptake into mitochondria	[159]

* VDAC: Voltage-Dependent Anion Channel; DCCD: *N*, *N*-dicyclohexylcarbodiimide; PTP: Permeability Transition Pore; Aacp: ADP/ATP carrier; MtCK: Mitochondrial Creatine Kinase; ER: Endoplasmic Reticulum.
